# Home Treatment of Consumption

**Published:** 1901-03

**Authors:** 


### Home Treatment of Consumption.

Osler believes that the arrest or cure of tuberculosis is a question entirely of nutrition, and the object of any treatment is to improve the physical condition of the patient that he can successfully withstand the attacks of the disease producing organisms. He advises that the patient spend a great deal of time in the open air, or if this be not possible, in a room with a southern exposure, with the windows wide open. The patient should be gradually accustomed to sleep with the windows open. As large quantities of good nutritious food as can be digested should be given; even overfeeding and stuffing should be practiced. Raw eggs are recommended, beginning with three per day and increasing one each week until one or two dozen are taken per day. He reports the case of a young woman who had well marked tuberculosis; her grandmother and two of her father's brothers had died of this disease. For more than a year she had had fever, had lost much in weight and had profuse night sweats. There were signs of extensive disease at the right apex. She was given special rules as to food and directed to spend most of the day in the open air, even when the weather was very cold. She began with three raw eggs per day and gradually increased to fifteen per day. Other good nutritious food was used with the eggs. At the end of eleven months she had gained twenty-three pounds and the cough and fever had disappeared, though there were still some moist râles at the right apex. No medicine was given except a cough mixture part of the time. Osler concludes with the following remark: "A rigid regimen, a life of rules and regulations, a dominant will on the part of the doctor, willing obedience on the part of the patient—these, with the conditions we have discussed, are necessary to the successful treatment of pulmonary tuberculosis."—*Public Health Journal.*

